# *Allophylus africanus* Stem Bark Extract Modulates the Mitochondrial Apoptotic Pathway in Human Stomach Cancer Cells

**DOI:** 10.3390/life13020406

**Published:** 2023-02-01

**Authors:** Vera Ribeiro, Federico Ferreres, Andreia Oliveira, Nelson G. M. Gomes, Ángel Gil-Izquierdo, Luísa Araújo, David Pereira, Patrícia Valentão

**Affiliations:** 1 REQUIMTE/LAQV, Laboratório de Farmacognosia, Departamento de Química, Faculdade de Farmácia, Universidade do Porto, R. Jorge Viterbo Ferreira, nº 228, 4050-313 Porto, Portugal; 2 Department of Food Technology and Nutrition, Molecular Recognition and Encapsulation (REM) Group, Universidad Católica de Murcia, UCAM, Campus Los Jerónimos, s/n, 30107 Murcia, Spain; 3 Research Group on Quality, Safety and Bioactivity of Plant Foods, Department of Food Science and Technology, CEBAS (CSIC), Campus University Espinardo, P.O. Box 164, 30100 Murcia, Spain; 4 MDS—Medicamentos e Diagnósticos em Saúde, Avenida dos Combatentes da Liberdade da Pátria, Bissau, Guinea-Bissau

**Keywords:** *Allophylus africanus*, Ethnopharmacology, isovitexin, Rhamnosylvitexin, Sapindaceae, vitexin

## Abstract

The present work aimed to detail the mechanisms elicited by *Allophylus africanus* P. Beauv. stem bark extract in human stomach cancer cells and to identify the bioactives underlying the cytotoxicity. MTT reduction and LDH leakage assays allowed characterizing the cytotoxic effects in AGS cells, which were further detailed by morphological analysis using phalloidin and Hoechst 33258. Proapoptotic mechanisms were elucidated through a mitochondrial membrane potential assay and by assessing the impact upon the activity of caspase-9 and -3. The extract displayed selective cytotoxicity against AGS cells. The absence of plasma membrane permeabilization, along with apoptotic body formation, suggested that pro-apoptotic effects triggered cell death. Intrinsic apoptosis pathway activation was verified, as mitochondrial membrane potential decrease and activation of caspase-9 and -3 were observed. HPLC-DAD profiling enabled the identification of two apigenin-di-*C*-glycosides, vicenin-2 (**1**) and apigenin-6*-C-*hexoside-8*-C-*pentoside (**3**), as well as three mono*-C-*glycosides-*O*-glycosylated derivatives, apigenin-7*-O-*hexoside-8*-C-*hexoside (**2**), apigenin-8*-C-*(2-rhamnosyl)hexoside (**4**) and apigenin-6*-C-*(2-rhamnosyl)hexoside (**5**). Isovitexin-2″*-O-*rhamnoside (**5**) is the main constituent, accounting for nearly 40% of the total quantifiable flavonoid content. Our results allowed us to establish the relationship between the presence of vicenin-2 and other apigenin derivatives with the contribution to the cytotoxic effects on the presented AGS cells. Our findings attest the anticancer potential of *A. africanus* stem bark against gastric adenocarcinoma, calling for studies to develop herbal-based products and/or the use of apigenin derivatives in chemotherapeutic drug development.

## 1. Introduction

Being one of the largest genera belonging to the Sapindaceae, *Allophylus* comprises more than 255 accepted species, many of which being known to have relevant effects that might be translated into the development of new herbal-based drugs and products [1]. Unlike the widely investigated *Allophylus edulis* (A.St.-Hil., A.Juss., and Cambess.) Radlk [2,3,4,5,6], other *Allophylus* spp., namely, *Allophylus africanus* P. Beauv. (Sapindaceae), remain scarcely investigated on their content in bioactives and biological effects.

*A. africanus* is the main African ‘species’, with a widespread and variable distribution growing in forest edges and open wood land, at altitudes from 960 to 1540 m, throughout a great part of Africa [7,8,9,10]. The leaves are reported to be frequently used in folk medicinal practices, namely, for the treatment of headaches and migraines, fever, rheumatic pain, diarrhea and several eye disorders [9,10,11,12,13]. Ethnomedicinal reports covering bark medicinal uses are less common, but it is worth mentioning its use as a vermicide, antimalarial, antiarthritic, and antidiarrheal agent [9,13,14,15]. Studies on the chemical characterization of *A. africanus* are basically limited to the fatty acid profiling of the leaves and flowers and their essential oils [16,17], isolation of the terpenes alloeudesmenol, hanocokinoside, allotaraxerolide, and alloaminoacetaldehyde also being described [12]. Characterization of the flavonoid profile of aqueous extracts obtained from the plant enabled the detection of 30 flavones, derived from apigenin and luteolin, on the leaves, apigenin derivatives being identified on the stem bark [18]. So far, *A. africanus* has been mainly investigated for its anti-inflammatory properties, with relevant effects being reported [10,18]. Previously, our group investigated the in vitro inhibitory effects of aqueous extracts obtained from the leaves and stem bark against 5-lipoxygenase and inflammatory mediators [18]. While the leaves appeared to be particularly effective in inhibiting the initiation of leukotrienes’ biosynthesis, both the leaves and stem bark extracts led to a significant reduction of NO levels in LPS-challenged RAW 264.7 macrophages [18]. Ibrahim and colleagues complemented our previous findings on the anti-inflammatory properties of *A. africanus*, demonstrating that a leaf extract significantly decreased carrageenan-induced oedema in rats [10].

While the plant has been mainly used in Africa to treat conditions with an inflammatory background [15,19], there are also ethnomedicinal reports indicating its use in cancer treatment in Tanzania [20], but nearly no experimental data on the in vitro or in vivo anticancer effects have been delivered. In this matter, it is worth referring the cytotoxic effects of a structurally new δ-tocotrienol obtained from the fruits of the plant, as well as three semi-synthetic derivatives, against human endocervical adenocarcinoma KB-3-1 cells [21].

A screening of 28 extracts obtained from a collection of plants from Guinea-Bissauan flora revealed a selective and strong cytotoxic effect towards human gastric AGS carcinoma cells upon treatment with a *A. africanus* stem bark extract [22]. Along with the scant data on the anticancer potential of the species, our preliminary findings prompted us to further investigate the events underlying the cytotoxicity of *A. africanus* stem bark against AGS cells through the elucidation of the anticancer mechanisms and the identification of the bioactives contributing to such effects.

## 2. Materials and Methods

### 2.1. General Chemicals, Reagents and Equipment

Sodium pyruvate, 3-(4,5-dimethylthiazolyl-2)-2,5-diphenyltetrazolium bromide (MTT), trypan blue, magnesium chloride, ethylenediaminetetraacetic acid (EDTA), Triton X-100, phalloidin–tetramethylrhodamine B isothiocyanate, Hoechst 33258, phorbol 12-myristate 13-acetate (PMA), dimethyl sulfoxide (DMSO), 3-[(3-cholamidopropyl)dimethylammonio1-propanesulfonate hydrate (CHAPS), sucrose, 1,4-dithiothreitol (DTT), and 2-[4-(2-hydroxyethyl)piperazin-1-yl]ethanesulfonic acid (HEPES) were obtained from Sigma-Aldrich (St Louis, MO, USA). Cell culture media, foetal bovine serum (FBS), and 1% penicillin/streptomycin (Pen Strep) were purchased from GIBCO, Invitrogen^TM^ (Gran Island, NY, USA). Human gastric AGS carcinoma cells were purchased from ATCC^®^, and human lung MRC-5 fibroblasts were purchased from Sigma-Aldrich (St Louis, MO, USA). Caspase-Glo^®^ 3/7 kit was purchased from Promega Corporation (Fitchburg, WI, USA), and caspase-9 substrate (Ac-Leu-Glu-His-Asp-7-amino-4-trifluoromethylcoumarin) was obtained from CPC Scientific (Sunnyvale, CA, USA). Staurosporine and 5,5′,6,6′-tetrachloro-1,1′,3,3′-tetraethylbenzimidazolylcarbocyanine iodide (JC-1) were purchased from Santa Cruz Biotechnology Inc (Heidelberg, Germany).

The cultures were kept in a humified CO_2_ incubator (Toreuse 2428, St. Louis, MO, USA). Spectrophotometric determinations were performed in a Cytation^TM3^ microplate reader. Morphological changes were recorded with a Nikon Eclipse Ts2R-FL microscope equipped with a Retiga R1 camera and a S Plan Fluor ELWD 20× DIC N1 objective, and images were analysed with a Fiji image processing package. HPLC-DAD analysis was performed with an analytical HPLC unit using a 150 mm × 4.6 mm, 5 μm Kinetex 100 Å RP-18 column (Phenomenex, Part No. 00F-4601-E0) coupled to Agilent 1260 series diode array detector (DAD) (Agilent Technologies) and controlled by Clarity software system, version 5.04.158 (DataApex, Ltd.).

### 2.2. Plant Material and Extraction

Stem bark from *A. africanus* was collected in October 2019 at Formosa Island, Guinea-Bissau. Voucher specimen (GB114) was deposited at the Faculty of Pharmacy, University of Porto. The sample was dried in a general protocol oven at 40 °C and sieved (mean particle size ≤910 μm). Dried plant material was extracted with a mixture of water and ethanol (1:1) using the conditions described in [22]. Extraction yield was 29.8% [22].

### 2.3. Cell-Based Assays

Human gastric AGS carcinoma cells and human lung MRC-5 fibroblasts were cultured in DMEM + GlutaMAx^TM^ and MEM + GlutaMAx^TM^ media, respectively, both supplemented with 10% FBS and 1% Pen Strep. Cultures were kept in a humified CO_2_ incubator set to 5% CO_2_ and 37 ºC. The dried hydroethanolic extract was redissolved in DMSO and diluted in culture medium to a maximum concentration of 0.125% to ensure the absence of cytotoxic effects.

### 2.4. Cell Viability and Membrane Integrity Assays

Effects of the hydroethanolic extract from *A. africanus* stem bark on the cell viability were evaluated through the MTT reduction assay [23]. Three independent experiments were performed in triplicate, and results were expressed as percentage of viability per concentration (μg mL^−1^) of extract relative to the negative control (culture medium).

The extracellular amounts of the cytosolic lactate dehydrogenase enzyme (LDH) were measured to determine the effects on the membrane integrity [23]. To ensure cell lysis, Triton X-100 (1% *v/v*) was used as positive control. Three independent experiments were performed in triplicate, and results correspond to the fold increase in absorbance recorded in treated vs. untreated cells.

### 2.5. Morphological Image Analysis

Morphological changes were investigated in AGS cells stained using phalloidin–tetramethylrhodamine B isothiocyanate and Hoechst 33258, both at 0.5 μg mL^−1^, during 20 min [22]. The ratio of cells with condensed/non-condensed chromatin were calculated considering, at least, four independent fields.

### 2.6. Caspase-3 and -9 Activity Assay

The evaluation of caspase-3 and -9 activity in AGS cells followed the procedure described in [24]. Briefly, caspase-3 activity was measured using the luminescent kits Caspase-Glo^®^ 3/7. Caspase-9 activity was evaluated with aliquots of cell extracts containing 50 µg of protein that were added to a reaction buffer composed of 25 mM HEPES, 0.1% CHAPS, 10% sucrose and 5 mM DTT, at a pH of 7.4. Staurosporine (0.25 µM) was used as a positive control.

### 2.7. Mitochondrial Membrane Potential Assay

To determine the mitochondrial membrane potential, AGS cells were seeded at a density of 15,000 cells well^−1^ in black bottom 96-well plates, in accordance with the procedure previously described [23]. Succinctly, prior to the addition of medium with JC-1 (7 μM) for 30 min, cells were pre-incubated with the extract at 250 µg mL^−1^ during 1 h. PMA (0.5 µM) was used as a positive control.

### 2.8. Phenolic Compounds Analysis by HPLC-DAD

The phenolic profile of the hydroethanolic extract from *A. africanus* stem bark (30 mg mL^−1^) was analyzed by HPLC-DAD, as described before [18]. Briefly, the solvent system was a gradient of water-formic acid (1%) (A) and acetonitrile (1%) (B). The elution started with 15% of B, and a gradient was used to obtain 30% of B at 20 min, and 50% of B at 30 min, at a flow rate of 0.8 mL min^−1^. Chromatograms were recorded at 340 nm, and data were processed on a Clarity software system, version 5.04.158. Quantification was achieved by calibration curves obtained with five different concentrations of external standards (in triplicate). Vicenin-2 (**1**) and its derivatives (**2** and **3**) were quantitated as vicenin-2, and 2″*-O-*rhamnosyl vitexin (**4**) and compound **5** were quantitated as vitexin-2″*-O-*rhamnoside.

### 2.9. Statistical Analysis

GraphPad Prism 6.01 Software (San Diego, CA, USA) was used to analyze the results obtained in the in vitro assays. Data were expressed as mean ± standard error of the mean (SEM) of, at least, three independent experiments, each performed in triplicate. The unpaired Student’s *t*-test was used to estimate the level of significance between each experimental condition relative to the control after identifying the outliers by the Grubbs’ test and verifying the normality of distribution by the Shapiro–Wilk test (differences at *p* ≤ 0.05 were considered significant).

## 3. Results

### 3.1. A. africanus Stem Bark Shows Selective Cytotoxicity in Human Gastric Adenocarcinoma AGS Cells

Our preliminary screening on the cytotoxicity of a collection of plant-derived extracts towards human lung A549 and human gastric AGS carcinoma cells suggested that *A. africanus* stem bark could elicit a selective cytotoxic response [22]. While no interference on the mitochondrial performance of A549 cells has been noted, the extract reduced the cell viability of AGS cells down to ca. 25% at 500 µg mL^−1^ [22].

**Figure 1:**
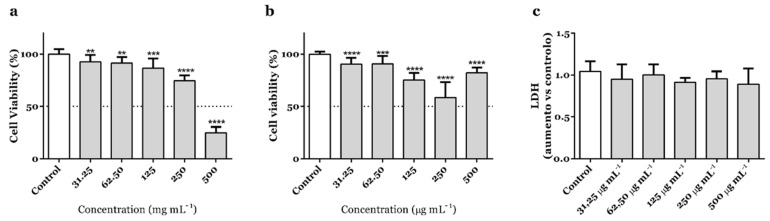
Effects on the cell viability of AGS (**a**) and MRC-5 cells (**b**) and on the LDH leakage of AGS cells (**c**) upon treatment with hydroethanolic extract from *A. africanus* stem bark. Results correspond to the mean ± standard error of the mean of three independent experiments, each performed in triplicate (statistical significance: ** *p* < 0.01, *** *p* < 0.001 and **** *p* < 0.0001).

The impact on the mitochondrial performance of adenocarcinoma AGS cells was now reinvestigated within a range of concentrations (31.25–500 µg mL^−1^) using an extract obtained from samples of *A. africanus* stem bark collected in a different location in Guinea-Bissau. Compared with untreated control cells, the extract significantly decreased the cell viability of AGS cells at the full range of concentrations, being worthwhile to note the reduction down to 24.9 and 74.5%, being recorded at 500 and 250 µg mL^−1^ (*p* < 0.0001), respectively (Figure 1a). The selectivity upon AGS cancer cells [22] is further suggested by the effects in human fetal lung fibroblast MRC-5 cells (Figure 1b). At 500 µg mL^−1^, *A. africanus* stem bark extract caused a reduction on the cell viability that was 3.3-fold-weaker (Figure 1b) than the one recorded in AGS cells at the same concentration (Figure 1a). However, while it was possible to observe that all concentrations caused a statistically significant change on the viability of MRC-5 cells, the effect was not concentration-dependent, differing from the effects upon AGS cells. Lactate dehydrogenase (LDH) release assay was used to elucidate if permeabilization of the plasma membrane occurred, i.e., potentially indicating necrotic cell death, but no significant effects have been recorded up to concentrations as high as 500 µg mL^−1^ (Figure 1c).

### 3.2. A. africanus Stem Bark Causes Apoptotic Cell Death in AGS Cells

**Figure 2:**
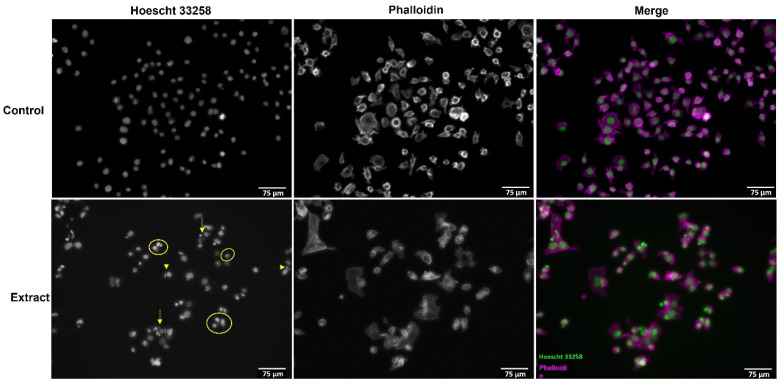
Morphological evaluation of the effect of the hydroethanolic extract from *A. africanus* stem bark on the AGS cell line after 24 h of incubation. The extract was tested at 500 µg mL^−1^. Overall cell morphology was assessed using phalloidin (cytoplasmic characteristics) and chromatin status (Hoechst 33258). Arrows—apoptotic bodies; circles—chromatin condensation.

To characterize the mechanism of cell death induced in AGS cells by *A. africanus* stem bark, treated and untreated cells were stained with phalloidin and Hoechst 33258 and observed under a fluorescence microscope. After exposure to the extract at 500 µg mL^−1^, for 24 h, there was an evident reduction on cell density in comparison with untreated cells (Figure 2). At the same time, the morphology of AGS cells changed significantly, with cell shrinkage and pyknosis (yellow arrows) being noted after treatment with the extract, and this allowed us to observe the effects after staining with Hoechst 33258 (Figure 2). While such morphological features, i.e., nuclear chromatin condensation, might occur due to necrosis or apoptosis [25,26], the use of a non-necrotic concentration and the apoptotic body formation (yellow circles) (Figure 2) suggest that the hydroethanolic extract obtained from *A. africanus* stem bark triggers apoptosis in AGS cells.

While no morphological changes have been detected to occur in the cytoplasm of AGS cells after the 24 h treatment with *A. africanus* stem bark extract (Figure 2), pyknosis is the main typical feature of apoptosis, followed by the production of cell fragments (apoptotic bodies) [26,27], with both characteristics being observed. While these morphological differences have been widely used to classify apoptosis, we aimed to elucidate if pro-apoptotic cell death was occurring via the extrinsic or the intrinsic pathway.

### 3.3. A. africanus Stem Bark Causes Caspase-Dependent Cell Death in AGS Cells via Activation of the Intrinsic Apoptosis Pathway

The altered mitochondrial membrane potential (MMP), and the subsequent release of cytochrome *c* and reactive oxygen species (ROS) to stimulate death signals, culminate in the initiation of the intrinsic apoptotic pathway [28]. As the mitochondrial apoptosis pathway acts as the primary regulator of endogenous apoptosis, being involved in the increase in outer membrane permeability, as well as change in MMP [28], potential interference of the extract under study was examined in AGS cells using JC-1 staining.

**Figure 3:**
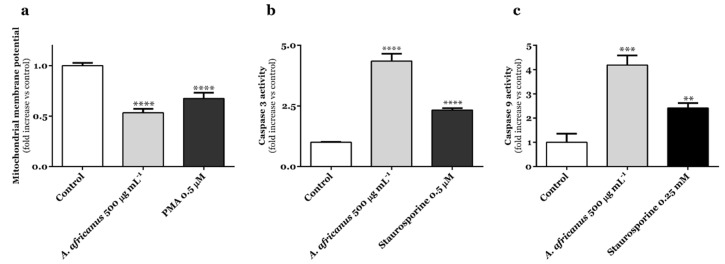
Effects of the hydroethanolic extract obtained from stem bark of *A. africanus* on the mitochondrial membrane potential (**a**) and in the activity of caspase-3 (**b**) and caspase-9 (**c**) in AGS cells, following 24 h of incubation at highest negative LDH concentration. Data represent the mean ± standard error of the mean of five independent experiments, each performed in triplicate (statistical significance: ** *p* < 0.01, *** *p* < 0.001 and **** *p* < 0.0001).

At 500 µg mL^−1^, *A. africanus* stem bark extract caused a significant decrease on MMP (*p* < 0.0001) in AGS cells, proving to be more potent than phorbol-12-myristate-13-acetate (PMA) at 0.5 µM (Figure 3a).

As the proteolytic maturation of caspase-9 and -3 is dependent on the allosteric activation of the apoptosis-protease activating factor 1 (Apaf-1) by the cytochrome *c* [29], caspase-3/9 activity was assessed in the treated and untreated AGS cells.

As shown in Figure 3b,c, the activities of caspases-3 and -9 in gastric adenocarcinoma AGS cells were greatly enhanced upon 24 h-treatment with the hydroethanol extract obtained from the stem bark of *A. africanus* (500 µg mL^−1^). It is worth noting that the recorded effects demonstrate a higher caspase activity than it was observed upon exposure to staurosporine (Figure 3b,c).

Results from the mitochondrial viability (MTT) assay show that *A. africanus* stem bark extract had a cancer-specific cytotoxic activity on the human gastric adenocarcinoma AGS cell line (Figure 1). The absence of effects upon LDH leakage (Figure 1c), along with the specific morphological features being observed upon Hoechst 33258 staining (Figure 2), strongly suggest that the cytotoxic effects elicited by the extract are related to the activation of pro-apoptotic pathways. In response to *A. africanus* stem bark extract, a significant loss of MMP was detected (Figure 3a), indicating the permeability transition of mitochondria, which subsequently leads to apoptosis. The activation of the initiator caspase-9 by *A. africanus* stem bark (Figure 3c) is suggested to trigger the activation of caspase-3, as confirmed in Figure 3b, largely supporting the activation of the intrinsic apoptosis pathway. The significant anticancer activity of *A. africanus* stem bark, herein demonstrated towards AGS cells, calls for the identification of the bioactives underlying or contributing to the effects.

### 3.4. Bioactive Phenolics in A. africanus Stem Bark Extract

**Figure 4:**
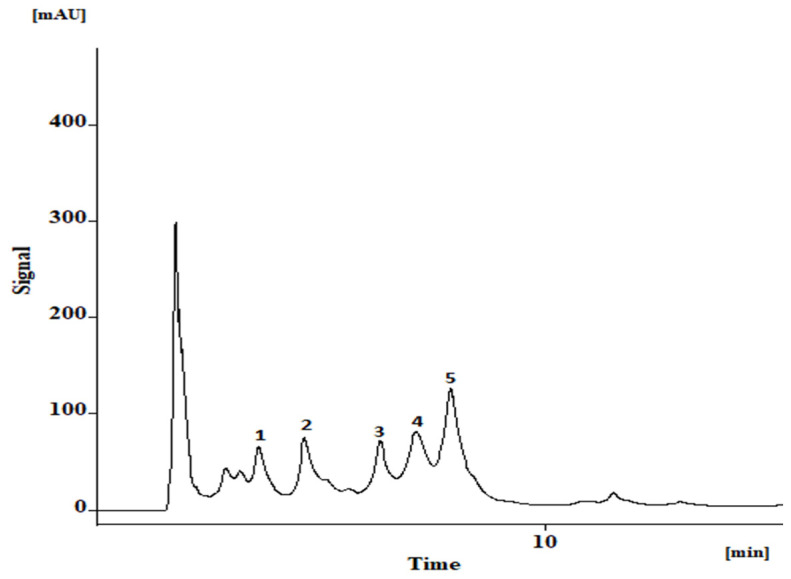
HPLC-DAD profile (340 nm) of the hydroethanolic extract from *A. africanus* stem bark. Compounds: (**1**) vicenin-2; (**2** and **3**) vicenin-2 derivatives; (**4**) 2″*-O-*rhamnosyl vitexin; and (**5**) 2″*-O-*rhamnosyl vitexin derivative.

The hydroethanol extract is characterized by the occurrence of the apigenin-di*-C-*glycosides, vicenin-2 (**1**), and apigenin-6*-C-*hexoside-8*-C-*pentoside (**3**), as well as the mono*-C-*glycosides*-O-*glycosylated derivatives, apigenin-7*-O-*hexoside-8*-C-*hexoside (**2**), apigenin-8*-C-*(2-rhamnosyl)hexoside (**4**), and apigenin-6*-C-*(2-rhamnosyl)hexoside (**5**) (Figure 4 and Table 1). Isovitexin-2″*-O-*rhamnoside (**5**) is the main constituent, accounting for nearly 40% of the total quantifiable flavonoid content (Table 1).

## 4. Discussion

So far, data on the anticancer effects of *Allophylus* spp. are mostly limited to preliminary screenings on the in vitro cytotoxicity against panels of cancer cell lines [30,31]. Specifically concerning to *A. africanus*, Biseko and collaborators demonstrated that organic extracts obtained from stem bark samples were particularly cytotoxic towards larynx carcinoma HEP-2 cells [20]. In analogy with our results, the cytotoxicity elicited by *A. africanus* stem bark appears to be selective, as no significant effects on the cell viability of hepatocellular carcinoma (HCC 1396) cells have been reported [20]. Our results suggest that the cytotoxicity caused by the extract obtained from the stem bark of *A. africanus* on AGS cells occurs through the intrinsic pathway of apoptosis, which involves permeabilization of the mitochondrial membrane to release cytochrome *c* into the cytosol. Subsequently, cytochrome *c* in the cytosol mediates Apaf-1 binding to pro-caspase-9, leading to caspase-9 activation [32].

While the leaves have been characterized on their content in terpenoids [12], fatty acids [16], and phenolic constituents [18], so far, only apigenin di*-C-*glycosides and mono*-C-*glycosides*-O-*glycosylated have been identified by us in an aqueous extract obtained from *A. africanus* stem bark [18]. Differently from our previous report [18], we have now characterized an hydroethanol extract obtained from stem bark samples of *A. africanus*, also collected in Guinea-Bissau, but in a different location (Formosa Island, in October 2019). Qualitatively, the hydroethanol extract did not differ from the previously profiled extract [18]. However, the HPLC-DAD analysis revealed a distinct quantitative profile, with isovitexin-2″*-O-*rhamnoside (**5**) being identified as the main constituent (Table 1), in contrast with the aqueous extract previously characterized, where apigenin-7*-O-*hexoside-8*-C-*hexoside (**2**) was the main flavonoid [18]. Unlike in our previous report, also the total quantifiable content was significantly lower in the hydroethanol stem bark extract (2741 ± 76 mg kg^−1^ dry extract; Table 1) in comparison with the aqueous extract (5141.75 ± 87.27 mg kg^−1^ dry extract) [18], which might be related with a different extraction efficiency enabled by water and ethanol, and/or the distinct phytogeographical conditions.

It is well known that the geographical region of plant habitat strongly impacts the qualitative and quantitative profiles of secondary metabolites, and that might frequently hamper the biochemical consistency and standardization of herbal products [33]. Flavonoid and phenolic profiles are not only dependent on genetic differences between the taxa, but also markedly affected by phytogeographical conditions such as temperature [34,35] and UV radiation [35]. In this matter, it is relevant to highlight that the overlapping qualitative profiles of the stem bark extracts of *A. africanus* (Table 1) [18], collected in different locations and during different periods, which evidence a chemically homogeneous raw material, thus enabling a consistent biological activity and the standardization of herbal products to be developed from the stem bark and based on the flavonoid profile.

*Allophylus* spp. have been reported on their content in flavonoid constituents, and they have been characterized by the occurrence of apigenin, cerarvensin, luteolin, myricetin, naringenin, and quercetin glycosides [4,18,36,37,38,39]. The apigenin-di*-C-*glycosides **1** and **3**, as well as the mono*-C-*glycosides*-O-*glycosylated derivatives **2**, **4,** and **5** (Table 1), have also been previously reported in the leaves of *A. africanus* [18]. Earlier, vicenin-2 (**1**) and vitexin-2″*-O-*rhamnoside (**4**) were described in samples of the leaves of *A. edulis* collected in Paraguay, along with the apigenin-mono*-C-*glycosides vitexin and isovitexin [4]. Additionally, Hoffmann-Bohm and colleagues allowed us to know the occurrence of **1**, **4**, and **5** and the apigenin-di*-C-*glycoside schaftoside on the leaves of *A. edulis var. edulis* and *A. edulis var. gracilis* [6], apigenin-8*-C-*β-rhamnopyranoside being later isolated from an extract obtained from *A. laevigatus* fruits [39].

The cytotoxic effects of *A. africanus* stem bark extract upon AGS cells appears to be mediated by the apigenin derivatives herein identified. While there are no reports on the anticancer effects of the main constituent occurring on the extract, i.e., isovitexin-2″*-O-*rhamnoside (**5**), neither on the 8-isomer **4**, the *C*-glycosyl flavones isovitexin and vitexin are known to be cytotoxic towards solid tumour cells, with such anticancer effects being mainly attributed to the activation of the intrinsic pathway through the upregulation of caspase-3 [40,41,42,43,44,45,46,47]. Indeed, both isovitexin and vitexin are particularly active on the induction of apoptosis in solid tumour cells, inducing the release of cytochrome *c* from the mitochondria to the cytosol and the loss of MMP [48,49]. Such events lead to the activation of the downstreaming caspase-3, as corroborated in HepG2 tumour-bearing mice upon treatment with isovitexin [48]. Specifically concerning human gastric cells, Zhou et al. [50] reported the effects of vitexin upon the gastric AGS cells with KRAS mutation, being also found to inhibit xenograft tumour growth and liver metastasis in vivo [50].

Closely related with vitexin-2″*-O-*rhamnoside (**4**), the diglycoside xylosylvitexin (vitexin-2″*-O-*xyloside) led to pro-apoptotic effects in human colon cancer RKO cells and a reduction of the S phase due to the specific arrest of the cell cycle in G1, effects that, according to Ninfali’s group [51], are in analogy with those observed with the aglycone apigenin [51].

While being detected as the constituent occurring in lowest amounts on *A. africanus* stem bark extract (Table 1), it is plausible to infer that vicenin-2 (**1**) contributes to the recorded activation of the intrinsic pathway in AGS cells (Figure 1a and Figure 3). Similarly to what we have found with *A. africanus* stem bark extract in AGS cells (Figure 3), activation of apoptosis by vicenin-2 (1) in the solid tumour cells NCI-H23 (non-small cell lung cancer) and HT-29 (human colon cancer) derives from the activation of caspase-3 [32,52], increased expression of cytochrome *c* being also reported to occur in the latter ones [52]. Yang and co-workers showed that vicenin-2 (50 µM) promotes substantial cell cycle arrest in the G2M phase and induces apoptosis in HT-29 cells, also increasing the expression of cytochrome *c*, Bax, and caspase-3 and suppressing the expression of Bcl-2 [52]. Vicenin-2 (**1**) also caused specific morphological changes in prostate carcinoma PC-3 and LNCaP cells, namely, chromatin clumping, as well as increased caspase-3 cleavage [53], which further corroborated the in vitro anticancer effects in mice bearing PC3 cells, vicenin-2 (**1**) being shown to induce potent regression of tumours and with PARP-cleavage being also recorded, thus supporting the pro-apoptotic effects of the apigenin derivative [53]. PARP cleavage induced by 1 is accompanied by decreased fibronectin levels, anti-apoptotic expression of Bcl2, and increased pro-apoptotic expression of Bax and tumor suppressor E-cadherin. The multiple effects of vicenin-2 (**1**) suggest its ability to modulate oncogenic, tumor suppressor, and differentiation cell signaling cascades to induce anticancer effects [53]. Recently, Li and colleagues described the in vivo effects of vicenin-2 (**1**; 30 mg kg^−1^), namely, the inhibition of Bcl-2 by triggering and/or activating apoptotic Bax in oral squamous cell carcinoma (OSCC) cell models [54]. Singhal and colleagues allowed us to know the synergistic effects of vicenin-2 (**1**), upon co-treatment with docetaxel in androgen-independent prostate cancer [55].

Current data on the apoptosis inducing effects of vicenin-2 (**1**), as well as on the apigenin derivatives that are closely structurally related with the diglycosides vitexin-2″*-O-*rhamnoside (**4**) and isovitexin-2″*-O-*rhamnoside (**5**) (Table 1), suggest that the effects of *A. africanus* stem bark upon AGS cells are partially mediated by the apigenin derivatives detected on the extract. However, the significant pro-apoptotic effects and the mechanisms underlying such properties might also be mediated by other classes of constituents occurring in *A. africanus* stem bark, certainly calling for additional investigational efforts to proceed with advanced pre-clinical studies and/or drug development.

## 5. Conclusions

In sum, in this study, we demonstrated that the cytotoxic effects of *A. africanus* stem bark upon gastric adenocarcinoma AGS cells are mediated by the activation of the intrinsic pathway of apoptosis, as suggested by the specific morphological changes and involving permeabilization of the mitochondrial membrane to release cytochrome *c* into the cytosol that are recognized as hallmarks of apoptosis and corroborated by the recorded activation of caspases-9 and -3. Considering that currently available data indicate that the apigenin derivatives identified on the extract, namely, vicenin-2 (**1**), contribute to the anti-AGS effects, and the qualitative profile in flavonoid bioactives remains unchanged with distinct phytogeographical conditions, as well as the development of a standardized herbal product with anticancer effects against gastric cancer is projected.

## Figures and Tables

**Table 1:** Phenolics content of the hydroethanolic extract from *A. africanus* stem bark ^a^.

Compounds		mg kg^−1^ Dry Extract
1	Vicenin-2	266 ± 40
2	Vicenin-2 derivative	461 ± 14
3	Vicenin-2 derivative	410 ± 17
4	2″*-O-*Rhamnosyl vitexin	523 ± 3
5	2″*-O-*Rhamnosyl vitexin derivative	1081 ± 2
	Total	2741 ± 76

^a^ Results correspond to mean ± standard deviation (*n* = 3).

## Data Availability

Data available from the corresponding author on reasonable request.

## Abbreviations

Apaf-1	Apoptotic protease activating factor-1
CHAPS	3-[(3-cholamidopropyl)dimethylammonium]-1-propanesulfonate
DAD	Diode array detector
DMSO	Dimethyl sulfoxide
DTT	1,4-dithiothreitol
EDTA	Ethylenediaminetetraacetic acid
FBS	Fetal bovine serum
HEPES	2-[4-(2-hydroxyethyl)piperazin-1-yl]ethanesulfonic acid
HPLC	High Performance Liquid Chromatography
IC_50_	Inhibitory concentration of 50%
LDH	Lactate dehydrogenase
MMP	Mitochondrial membrane potential
MTT	3-(4,5-dimethylthiazolyl-2)-2,5-diphenyltetrazolium bromide
NCI-H23	Human non-small cell lung cancer
OSCC	Oral squamous cell carcinoma
PARP	Polymerase Poly(ADP-Ribose)
PMA	Phorbol 12-myristate 13-acetate
ROS	Reactive oxygen species
Rt	Retention time
